# Clinical Significance of Fecal Calprotectin for Evaluating Mucosal Inflammation with IgA Vasculitis

**DOI:** 10.31662/jmaj.2021-0173

**Published:** 2022-03-25

**Authors:** Riko Kato, Masato Oguri, Shinichi Tsubata, Yuichi Adachi

**Affiliations:** 1 Department of Pediatrics, Toyama Red Cross Hospital, Toyama, Japan; 2 Department of Pediatrics, Faculty of Medicine, University of Toyama, Toyama, Japan

**Keywords:** IgA vasculitis, alpha _1_-antitrypsin, fecal calprotectin, fecal immunochemical test, inflammatory bowel disease

## Abstract

IgA vasculitis is the most common systemic small vasculitis in children. Its major clinical manifestations are palpable purpura, arthritis and arthralgias, gastrointestinal involvement, and renal manifestations. Regarding gastrointestinal manifestations, steroids are effective in reducing abdominal pain. However, exacerbation of gastrointestinal manifestation is frequently experienced when the steroid dose is being tapered. Thus, reliable biomarkers for gastrointestinal mucosal inflammation are needed. We report the case of a 4-year-old girl with abdominal-type IgA vasculitis. During the clinical course, we used several markers, such as fecal immunochemical test, fecal α_1_-antitrypsin and calprotectin. When fecal immunochemical test showed negative results and fecal α_1_-antitrypsin value returned to the normal range, corresponding to her abdominal pain improvement, fecal calprotectin levels remained high. This suggests that fecal calprotectin is more sensitive for evaluating mucosal inflammation than other markers. It could be a useful marker for mucosal inflammation in IgA vasculitis.

## Introduction

IgA vasculitis (IgAV) is the most common systemic small vasculitis in children. Palpable purpura and gastrointestinal (GI) involvement, arthritis, and renal involvement are major clinical manifestations. GI symptoms are seen in 50%-75% of patients ^(1)^. Although corticosteroids effectively reduce GI symptoms, there are no guidelines recommending the dose and duration of steroids. Patients are often treated empirically and experience recurrence of symptoms when the steroid dose is being tapered. Therefore, reliable biomarkers for GI mucosal inflammation are needed. Fecal calprotectin (FC) and fecal immunochemical test (FIT) have been widely used to monitor mucosal inflammation in inflammatory bowel diseases (IBD)^(2), (3)^. We examined whether these markers could be used to evaluate GI mucosal inflammation while tapering steroids in a child with IgAV.

## Case Report

**Figure 1.**
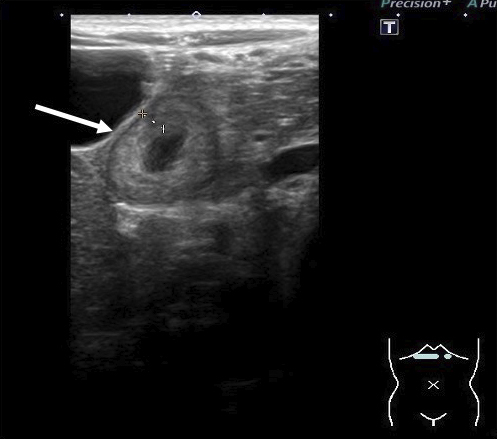
Abdominal ultrasound. The white arrow indicates wall thickening (4.1 mm) of the duodenum.

A 4-year-old girl was admitted with persistent abdominal pain and vomiting. A referring physician gave the patient probiotics; however, her symptoms did not improve. The patient had upper abdominal pain without guarding but did not have palpable purpura or arthritis. Laboratory results showed a white blood cell (WBC) count of 23.2 × 10^3^/μL (reference range, 5.5-15.5 × 10^3^/μL) and a C-reactive protein (CRP) level of 6.70 mg/dL (reference range, <0.3 mg/dL). Urine occult blood and protein levels were negative. An abdominal ultrasound showed marked wall thickening (4.1 mm) of the duodenum (Figure 1). Despite antibiotic treatment, the patient’s abdominal symptoms continued. On posthospitalization day 3, laboratory tests revealed a higher fibrin/fibrinogen degradation products level (24.2 μg/mL; reference range, <5.0 μg/mL) and decreased blood coagulation factor ⅩIII activity (34%; reference range, 70%-140%). We diagnosed the patient with abdominal-type IgAV i.e., only abdominal symptoms with no other clinical manifestations, and started to treat her with prednisolone (2 mg/kg/day).

Her abdominal pain promptly improved after starting prednisolone, and an abdominal ultrasound showed improved duodenal wall thickness (DWT). After a 3-day course of prednisolone, we began tapering the dose from 2 mg/kg/day to 1 mg/kg/day. However, abdominal pain recurred on the first night of tapering; thus, we increased the dose back to 2 mg/kg/day. The steroid was gradually tapered over 30 days, and she had no recurrent abdominal pain.

**Figure 2.**
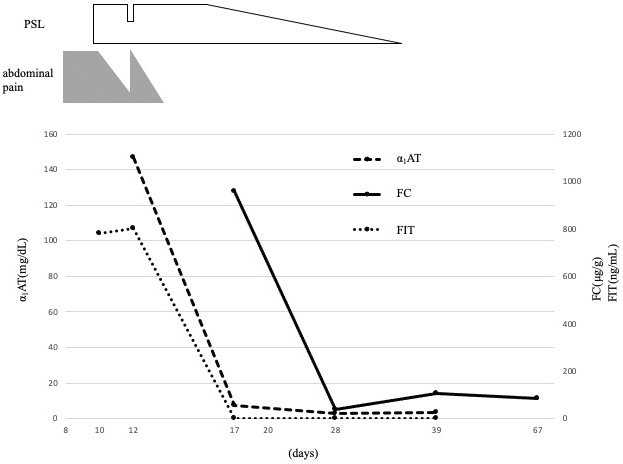
Time course of clinical symptoms and fecal biomarkers. α_1_-AT: alpha_1_-antitrypsin (reference range, <10.0), FC: fecal calprotectin (reference range, <239.9), FIT: fecal immunochemical test, PSL: prednisolone.

To evaluate which test is most reliable for assessing GI mucosal inflammation in IgAV, we measured the FC, FIT, and fecal α_1_-antitrypsin (α_1_-AT) several times during the clinical course. During the first 7 days after hospitalization, even when the abdominal pain was initially improved by prednisolone, the levels of several markers, such as WBC, CRP, and α_1_-AT, remained high, and FIT was positive. After increasing the prednisolone dose again, her symptoms improved, and the WBC, CRP, and α_1_-AT levels returned to normal ranges. However, the FC level remained high (Figure 2). On day 21 of hospitalization, all markers, including FC, returned to normal ranges.

## Discussion

Although the palpable purpura is essential for diagnosing IgAV, it is not always the initial manifestation ^(1)^. Some cases were reported to have only GI symptoms without any purpura at their clinical course ^(4), (5)^. The European League against Rheumatism and Paediatric Rheumatology European Society recommends histological examination for the diagnosis of IgAV ^(6)^. We could not perform GI endoscopy because it was invasive for young children, so we had no histological diagnosis. However, it is reported that imaging studies and blood coagulation tests are helpful in identifying the thickening of the gastrointestinal wall, especially in the duodenum, and decreased coagulation factor ⅩIII activity in IgAV, respectively ^(7), (8)^. In our case, considering DWT and decreased coagulation factor XIII, we diagnosed the patient with abdominal-type IgAV.

FC, a mucosal inflammation marker for IBD, is a calcium-binding cytosolic protein located mainly in neutrophils and macrophages ^(2), (3), (9)^. It has been reported that FC increases in several acute abdominal diseases ^(9)^. There are few reports evaluating the clinical significance of FC in abdominal-type IgAV ^(9), (10)^. Teng et al. showed that an increase of FC in the early stage of acute phase IgAV tended to decrease after remission, reflecting the extent of intestinal inflammation ^(10)^. As a new finding in our case, FC concentration remained high even when the patient’s abdominal pain and DWT improved, and FIT and α_1_-AT became negative. After the FC level normalized, we could discontinue the steroid without recurrence of abdominal symptoms. This suggests that FC is more sensitive for evaluating mucosal inflammation than FIT and α_1_-AT. Further studies are warranted for its clinical application while tapering the corticosteroid.

## Article Information

### Conflicts of Interest

None

### Acknowledgement

We thank our patient and her parents for their consent to publish this case report. We also thank the radiologists for their support in the diagnosis and treatment.

### Author Contributions

R.K. designed this case report and drafted the initial manuscript. M.O. and S.T. provided technical support and conceptual advice. Y.A. reviewed and supervised the manuscript.

All authors have approved the final manuscript and agree to be accountable for all aspects of the manuscript.

### Approval by Institutional Review Board (IRB)

This manuscript is a case report and does not need approval by IRB.

### Informed Consent

The patient and parents of the patient are aware of the intent to publish this case report and agree to it.
